# Maternal and Neonatal Outcomes of Pregnant Women with Abnormal 50 g Glucose Challenge Tests in Nakhon Si Thammarat, Thailand: A Retrospective Study

**DOI:** 10.3390/ijerph20227038

**Published:** 2023-11-08

**Authors:** Suda Jaihow, Nonthapan Phasuk, Udomsak Narkkul, Wipawan Chaoum Pensuksan, Stephen J. Scholand, Chuchard Punsawad

**Affiliations:** 1 School of Nursing, Walailak University, Nakhon Si Thammarat 80160, Thailand; sudajaihow@gmail.com; 2 Department of Medical Clinical Sciences, School of Medicine, Walailak University, Nakhon Si Thammarat 80160, Thailand; nonthapan.ph@wu.ac.th; 3 Research Center in Tropical Pathobiology, Walailak University, Nakhon Si Thammarat 80160, Thailand; udomsak.na@wu.ac.th; 4 Department of Medical Sciences, School of Medicine, Walailak University, Nakhon Si Thammarat 80160, Thailand; 5 Faculty of Nursing, Suratthani Rajabhat University, Surat Thani 84100, Thailand; wipawan.pen@sru.ac.th; 6 Department of Medicine, University of Arizona, Tucson, AZ 85724, USA; rabiesfreeworld@yahoo.com

**Keywords:** maternal outcome, neonatal outcomes, pregnant women, glucose challenge test

## Abstract

(1) Background: An abnormal 50 g glucose challenge test (50 g GCT) during pregnancy, even without a diagnosis of gestational diabetes mellitus (GDM), may result in undesirable obstetric and neonatal outcomes. This study sought to evaluate the outcomes in pregnant women with abnormal 50 g GCT in secondary care hospitals in Thailand. (2) Methods: A total of 1129 cases of pregnant women with abnormal 50 g GCT results who delivered between January 2018 and December 2020 at Thasala, Sichon, and Thungsong hospitals were retrospectively reviewed and divided into three groups: abnormal 50 g GCT and normal 100 g oral OGTT (Group 1; n = 397 cases), abnormal 50 g GCT and one abnormal 100 g OGTT value (Group 2; n = 452 cases), and GDM (Group 3; n = 307 cases). (3) Results: Cesarean section rates in group 3 (61.9%) were statistically higher than those in groups 1 (43.6%) and 2 (49.4%) (*p* < 0.001). In addition, the highest rate of birth asphyxia was found in group 2 (5.9%), which was significantly higher than that in Groups 1 (1.8%) and 3 (3.3%) (*p* = 0.007). (4) Conclusions: Pregnant women with abnormal 50 g GCTs without a diagnosis of GDM had undesirable maternal and neonatal outcomes, as well as those who had GDM, suggesting that healthcare providers should closely monitor them throughout pregnancy and the postpartum period.

## 1. Introduction

An abnormal test is associated with a higher risk of gestational diabetes mellitus (GDM), independently of whether it resolves or not after delivery. It is usually diagnosed after 20 weeks of gestation [1]. There is increasing evidence on the long-term health outcomes of mothers with GDM and their offspring [2]. There is no current national guideline for GDM screening in Thailand. In general, most hospitals follow the guidelines of Siriraj Hospital, one of the quaternary university hospitals in Thailand [3]. According to this guideline, pregnant women without risk factors are screened using the 50 g GCT test at 24–28 weeks of gestation, while those with risk factors, including age ≥30 years; pre-pregnancy body mass index (BMI) ≥ 25 kg/m^2^; family history of diabetes; presence of hypertension; previous GDM; and a history of fetal macrosomia, stillbirth, or fetal anomaly are screened at their first antenatal care visit and again at 24–28 weeks of gestation. If the results exceed the screening threshold, then they undergo a 100 g oral glucose tolerance test (OGTT) for GDM diagnosis. [4]. The American College of Obstetricians and Gynecologists recommends that all pregnant women should undergo a two-step method to screen and diagnose GDM [5], which uses an initial 50 g GCT for the screening test followed by a diagnostic 100 g oral glucose tolerance test (OGTT) if the 50 g GCT is positive (≥140 mg/dL). According to the International Association of Diabetes and Pregnancy Study Groups (IADPSG) criteria, a one-step method uses only a 75 g OGTT as a diagnostic test for GDM [6,7]. GDM is defined as the presence of two or more positive values, and impaired glucose tolerance is defined as positive for a single value on the 100 g OGTT [8,9] or the 75 g OGTT [10].

The prevalence of GDM has increased dramatically. The highest prevalence of GDM (24.2%) has been found in Southeast Asia [11]. In Asia, the prevalence of GDM is 11.5%. In Thailand, the incidence rates of GDM diagnosed before 20 weeks and at 24–28 weeks of gestation are 14% and 15%, respectively [3,12]. Apart from GDM, a previous study reported that the incidence of abnormal 50 g GCTs in pregnant women increased from 10.6% in 2005 to 18.1% and from 20.2% to 31.3% in those with high normal GCT (>125 mg/dL) [13]. In Thailand, 37.4% of pregnant women had a positive 50 g GCT result [3]. A previous report documented that women with a positive 50 g GCT and negative diagnostic tests (normal 100-g OGTT) have an increased risk of adverse pregnancy outcomes, including macrosomia, cesarean delivery, preeclampsia, and shoulder dystocia [14]. Women with a 50 g GCT > 200 mg/dL have been associated with preeclampsia and shoulder dystocia [15], preterm birth, cesarean delivery, postpartum hemorrhage, increased risk of cardiovascular diseases [16], respiratory morbidity, neonatal hypoglycemia, and babies who are large for their gestational age (LGA) [17,18]. The 2020 Standards of Medical Care in Diabetes include current clinical practice recommendations for diabetes, but do not address the issue of managing pregnant women with abnormal 50 g GCTs [1]. 

In Thailand, there are no guidelines on standard care for pregnant women with abnormal 50 g GCTs. Furthermore, there is still a lack of study data regarding the outcomes in pregnant women with abnormal 50 g GCTs who do not have diagnoses of GDM. There is still a lack of study data regarding the outcomes in pregnant women with abnormal 50 g GCTs without diagnoses of GDM. There are different contexts and guidelines for GDM management among institutions. In Thailand, previous studies conducted in tertiary hospitals in urban areas have reported the effects and risk factors of abnormal GDM screening [19,20], but the data in primary health care units and community hospitals are less clear. There are differing guidelines for the management of GDM among institutions. Therefore, this study aimed to examine the impact of abnormal 50 g GCT on maternal and neonatal outcomes in three main secondary care hospitals in Nakhon Si Thammarat Province, south of Thailand, and to compare these outcomes among women with abnormal 50 g GCTs with normal 100 g OGTTs, pregnant women with abnormal 50 g GCTs and one abnormal 100 g OGTT result, and pregnant women with GDM.

## 2. Materials and Methods

### 2.1. Study Design and Setting

**Figure 1:**
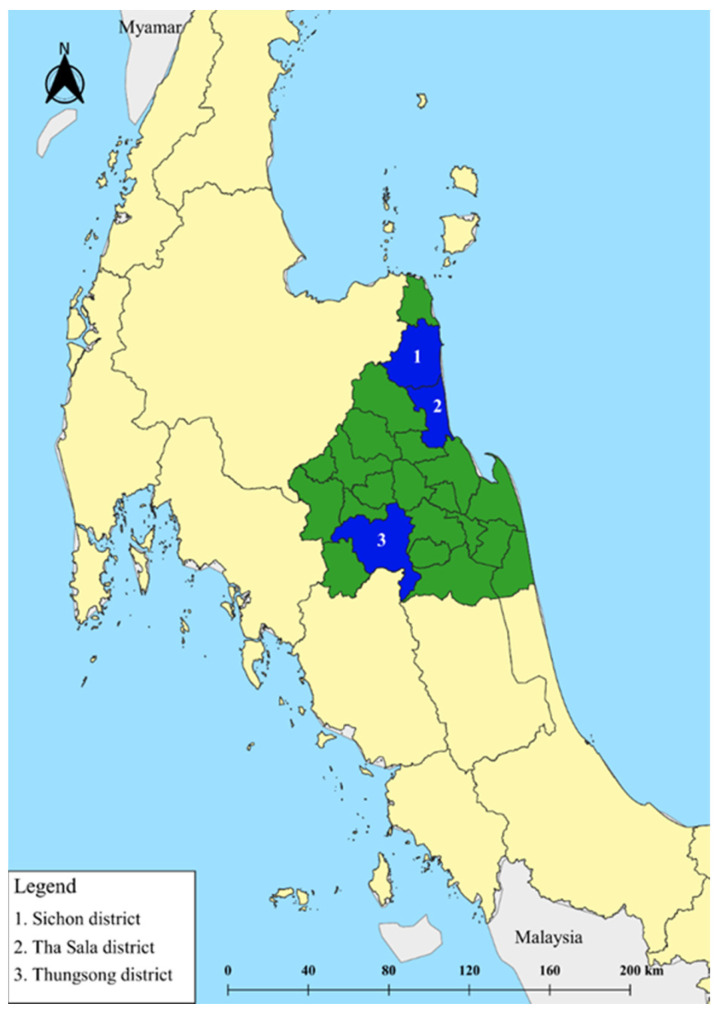
Location of the study sites—Sichon (1), Thasala (2), and Thungsong (3) District, Nakhon Si Thammarat Province, Thailand. Quantum GIS version 3.16.11 (ESRI base maps) was used to generate the map (https://qgis.org/en/site/ (accessed on 6 June 2022)).

This retrospective study was carried out at three community hospitals (Sichon, Thasala, and Thungsong) located in Nakhon Si Thammarat Province, Thailand. At these hospitals, approximately 120–250 pregnant women per month use antenatal care and delivery services, and there are 1–3 obstetricians, 2–3 midwives in the antenatal care unit, and 8–10 midwives in the labor room. Sichon, Thasala, and Thungsong Hospitals are located in Sichon District (9°0′24″ N latitude and 99°54′6″ E longitude, approximately 714 km south of Thailand’s capital), Thasala District (8°40′0″ N latitude and 99°55′54″ E longitude, approximately 752 km south of Thailand’s capital), and Thungsong District (8°9′53″ N latitude and 99°40′51″ E longitude, approximately 765 km south of Thailand’s capital), respectively (Figure 1).

### 2.2. Participants, Sample Size, and Group Classification

Pregnant women with abnormal 50 g GCTs who had attended antenatal clinics and delivered between January 2018 and December 2020 at Thasala, Sichon, and Thungsong Hospitals were enrolled in this study. The sample size was determined using the single-proportion population formula:

n = N/(1 + N*e*^2^) (1)

where n is the sample size, N is the population size (Sichon Hospital = 3780, Thasala Hospital = 4320, and Thungsong Hospital = 5400), and *e* is the acceptable sampling error (0.05). The confidence level was maintained at 95%. The sample size from the three hospitals was 1100. We determined the prevention of missing data based on a sample size of 5%. Therefore, the final sample size was 1155 participants. However, only 1129 complete medication records were included in this study.

The inclusion criteria were women with singleton pregnancies, who had undergone GDM screening by 50 g GCT at 24–28 weeks of gestation, and who had attended and delivered at Sichon, Thasala, and Thungsong Hospitals between January 2018 and December 2020. The exclusion criteria were pregestational diabetes, gestational hypertension, preeclampsia, chronic hypertension, and incomplete medical data records. Finally, a stratified random sampling method was used for data collection. In this study, the pregnant women were classified into three groups depending on the number of abnormal 100 g OGTT values: group 1 consisted of pregnant women who had abnormal 50 g GCTs and normal 100 g OGTTs; group 2, pregnant women with abnormal 50 g GCTs and one abnormal 100 g OGTT, who had a GCT value ≥140 mg/dL and were positive for a single 100 g OGTT; and group 3, pregnant women with GDM who had been diagnosed with a GCT value ≥140 mg/dL and who had two or more positive 100 g OGTT values. The diagnosis of GDM was based on the Carpenter–Coustan criteria, and was based on the 100 g OGTT. GDM was defined as having any two values at or above thresholds of 95 mg/dL, 180 mg/dL, 155 mg/dL, and 140 mg/dL for fasting, 1 h, 2 h, and 3 h plasma glucose levels, respectively [21].

### 2.3. Data Source and Collection

Retrospective data were extracted from the HOSxP program and medical records (antenatal and delivery records). There were three parts, including (1) the baseline maternal characteristics, such as age, religion, past pregnancy history, risk factors for GDM (age ≥30 years; pre-pregnancy BMI ≥ 25 kg/m^2^; family history of diabetes; presence of hypertension; previous GDM; and a history of fetal macrosomia, stillbirth, or fetal anomaly) [4], gravidity and parity, disease or complication during pregnancy, pre-pregnancy weight, height, BMI, gestational weight gain, diabetes in a 1st degree family member, blood pressure at delivery, 50 g GCT value, 100 g OGTT value, total number of antenatal care visits, and gestation at first antenatal care visit; (2) neonatal outcomes, such as gestational age at delivery, prematurity (delivery before 37 weeks of gestational age), sex, birth weight, LGA (infants whose weight was above the 90th percentile for gestational age), macrosomia (a fetus larger than 4000 g), neonatal hypoglycemia (glucose < 40 mg/dL in the first 24 h after birth) [22], Apgar score at 1st and 5th minute, admission into neonatal intensive care unit, birth asphyxia (defined as mild and moderate birth asphyxia with 1 min Apgar score 4–7 and severe birth asphyxia with 1 min Apgar score 0–3 [23]), heart rate, respiration rate, oxygen saturation, and blood sugar; and (3) maternal outcomes such as mode of delivery, shoulder dystocia, and total blood loss.

### 2.4. Ethical Considerations

This study was reviewed and approved by the Ethics Committee on Human Research at Walailak University, Thailand. This study was performed in accordance with the Declaration of Helsinki (Certificate of Ethical Approval number: WUEC-21-025-01). The requirement for written consent documentation was waived due to the unidentifiable personal information in the secondary data.

### 2.5. Statistical Analysis

The data were analyzed using SPSS for Microsoft Windows (version 22.0; IBM, Armonk, NY, USA). Descriptive statistics were used to analyze the demographic data and maternal and neonatal outcomes. Continuous variables are presented as medians and interquartile ranges (IQRs), and categorical variables are presented as frequencies and percentages. The Kolmogorov–Smirnov test was used to check the normality of the continuous data. Regarding the continuous variables, the Kruskal–Wallis and Mann–Whitney U tests were applied to compare the median of data among more than two groups and two groups, respectively. For categorical variables, the chi-square test was used to examine bivariate associations between demographic data and maternal and neonatal outcomes among the groups. Statistical significance was set at a *p*-value < 0.05.

## 3. Results

### 3.1. Characteristics of Pregnant Women with Abnormal 50 g GCT

Table 1 shows the demographic characteristics of pregnant women with abnormal 50 g GCTs from the three secondary care hospitals. The median maternal age was 31 (26–35) years, and 41.5% had a family history of diabetes. Of the study participants, 875 (75.5%) were multiparous and 898 (79.5%) were Buddhists. The prevalence of Islam and pre-pregnancy BMI > 25 kg/m^2^ at Thasala Hospital were higher than those at Sichon and Thungsong Hospitals. Of these, 397 (35.2%) were pregnant women with normal 100 g OGTTs; 425 (37.6%) were pregnant women with one abnormal 100 g OGTT value; and 307 (27.2%) had been diagnosed with GDM.

### 3.2. Maternal and Neonatal Outcomes of Pregnant Women with Abnormal 50 g GCT

The most common maternal outcome was cesarean section (50.8%, 573/1129), followed by postpartum hemorrhage (4.2%, 47/1129) and shoulder dystocia (3.4%, 38/1129). The rates of postpartum hemorrhage and shoulder dystocia at Thasala Hospital were higher than those at Sichon and Thungsong Hospitals. The most common neonatal outcome was hypoglycemia (37%, 232/1129), followed by LGA (35.6%, 402/1129) and respiratory distress syndrome (33.9%, 383/1129). The rates of macrosomia and LGA were higher at Thasala Hospitals than at Sichon and Thungsong Hospitals. In addition, the rates of respiratory distress syndrome at Sichon Hospital were higher than those at Thasala and Thungsong Hospitals (Table 2).

### 3.3. Comparison of Demographic Characteristics among Groups

The median maternal age in group 3, the GDM group (33; 29–37 years), was significantly higher than that in groups 1 (30; 26–34 years) and 2 (31; 26–35 years) (*p* < 0.001). The median pre-pregnancy BMI, systolic blood pressure, and previous GDM in group 3 were significantly higher than those in groups 1 and 2 (all *p* < 0.05) (Table 3). Group 3 had a higher percentage of pregnant women with BMIs greater than 25 kg/m^2^ than groups 1 and 2 (*p* < 0.001). There was no statistically significant difference between the groups regarding family history of DM, history of macrosomia, or rate of total weight gain greater than the recommendation (Table 3).

### 3.4. Maternal and Neonatal Outcomes with Comparison among Groups

Table 4 shows a comparison of the maternal and neonatal outcomes among the groups. The rates of cesarean section in group 3 (61.9%) were statistically higher than those in groups 1 (43.6%) and 2 (49.4%) (*p* < 0.001). Nonetheless, there were no statistically significant differences in the rates of shoulder dystocia or postpartum hemorrhage among the groups. Regarding the neonatal outcomes, the highest rate of birth asphyxia was found in group 2 (5.9%), which was statistically higher than that in groups 1 (1.8%) and 3 (3.3%) (*p* = 0.007). The rates of neonatal intensive care unit admission and hypoglycemia in group 3 were significantly higher than those in groups 1 and 2. The median neonatal birth weight, preterm birth, macrosomia, LGA, and rates of respiratory distress syndrome were comparable between the groups.

## 4. Discussion

This study was designed to compare the maternal and neonatal outcomes among pregnant women who had an abnormal 50 g GCTs with normal 100 g OGTT, pregnant women with abnormal 50 g GCTs and one abnormal 100 g OGTT value, and pregnant women with GDM. The majority of the pregnant women in secondary care hospitals in Nakhon Si Thammarat province in southern Thailand were positive for 50 g GCT and negative for 100 g OGTT, aged more than 25 years, and multigravida, and had family histories of DM, GDM risks of more than one factor, total weight gain within the recommendation, and vaginal deliveries. Similarly to previous studies in large tertiary hospitals in Bangkok, Thailand, the results showed that the majority of pregnant women with abnormal GCT were aged ≥25 years and multigravida [3,19,20]. As for Japanese women who had false positive GCT results at 24–28 weeks of gestation, the mean age was 31.4 ± 5.5 years, with 43.3% nulliparity [25]. Moreover, there are several factors associated with an abnormal 50 g GCT, such as a family history of diabetes, prior macrosomia, and a history of spontaneous abortions [26,27]. The pregnant women with positive 50 g GCTs were significantly older and had higher pre-pregnancy BMIs (kg/m^2^) [28]. Some studies have also reported a total weight gain greater than recommended among this group of women [29]. In the present study, the results showed that a history of GDM in previous pregnancies was more common in GDM cases, but there was no difference between the normal 100 g OGTT group and group with one abnormal 100 g OGTT value, which was consistent with a previous report in Thailand [3]. However, pregnant women with GDM were found to be significantly older and had higher pre-pregnancy BMIs, higher blood pressure, GDM risks of more than one factor, and previous GDM than those who had abnormal 50 g GCTs without GDM. GDM was significantly associated with age >35 years [30], which is similar to the results of our study. 

Regarding maternal outcomes, the results showed that the most common maternal outcome was cesarean delivery (50.8%). The cesarean section rate in this study was comparable to the rates of cesarean delivery in Thailand in the previous report [31]. The rates of cesarean section were statistically higher in GDM cases than in the groups with abnormal 50 g GCTs and normal 100 g OGTTs, or those with one abnormal 100 g OGTT value. Nonetheless, there were no statistically significant differences in the rates of shoulder dystocia and postpartum hemorrhage among the groups. These results can be explained by the fact that women with abnormal 50 g GCTs and GDM had high blood sugar levels and fetal exposure to hyperglycemia throughout pregnancy. Hyperglycemia leads to an increase in fetal fat and protein stores, resulting in macrosomia [32]. GDM has clear management practice guidelines. Therefore, healthcare providers should closely monitor pregnant women and newborns in this group. In Thailand, elective caesarean sections were usually performed to prevent maternal and neonatal trauma. However, there is no current recommendation for elective cesarean section in mothers with abnormal 50 g GCTs without diagnoses of GDM [1]. Additionally, there was no statistically significant difference in the rates of shoulder dystocia or postpartum hemorrhage among the groups. This suggests that, even without a diagnosis of GDM, the outcomes (shoulder dystocia and postpartum hemorrhage) were not different from those who had GDM. 

With regard to neonatal outcomes, the most important finding in this study was the prevalence of birth asphyxia in pregnant women who had abnormal 50 g GCTs and one abnormal 100 g OGTT value, which was statistically higher than that in the abnormal 50 g GCT, normal 100 g OGTT, and GDM groups. In this study, women with abnormal 50 g GCTs and one abnormal 100 g OGTT value had a 3.48 times increased risk of delivering infants with birth asphyxia compared to those with abnormal 50 g GCTs only (95% confidence interval: 1.48–8.14). This is inconsistent with previous research that found the frequency of birth asphyxia to be significantly higher in the infants of mothers with prior diabetes (22%) and GDM (20%) than those with non-diabetic mothers (6%) [33]. These results may be due to the studies having been conducted in secondary care hospitals. These hospitals do not have intensive units for the management of GDM or standard recommendations or practice guidelines to provide care for women with positive 50 g GCTs who have not been diagnosed with GDM [1], thus resulting in a higher rate of birth asphyxia. In contrast, GDM has standard clinical practice guidelines. Pregnant women with GDM are closely monitored by healthcare providers from antenatal care to the postpartum period.

The other important findings of this study were the comparable rates of neonatal birth weight, preterm birth, macrosomia, LGA, and rates of respiratory distress syndrome among pregnant women with GDM and pregnant women with abnormal 50 g GCTs and without GDM. Pregnant women with abnormal 50 g GCTs without diagnoses of GDM may also deliver LGA babies with macrosomia. Hyperglycemia can lead to fetal exposure to hyperglycemia during pregnancy. Hyperglycemia leads to an increase in fat and protein stores in the fetus. Most LGA babies have maternal hyperglycemia [25,28,34]. LGA is associated with several adverse outcomes, including elevated rates of neonatal hypoglycemia, shoulder dystocia, and a prolonged labor process [34]. Women with positive 50 g GCT and negative 100 g OGTT results were considered to have similar adverse outcomes to those with GDM. To prevent negative effects on maternal and neonatal health, we suggest that healthcare providers carefully follow up on these pregnant women. 

The rates of neonatal intensive care unit admission and hypoglycemia in the GDM group were statistically higher than in the group of pregnant women who had abnormal 50 g GCTs without a diagnosis of GDM (*p* < 0.001). These findings, therefore, confirmed the previous results that indicated that neonatal hypoglycemia was more common in pregnancies with GDM [35]. Similarly, hypoglycemia developed in 38.3% of infants of diabetic mothers, and 43.2% of mothers with neonatal hypoglycemia had GDM [36]. Neonatal hypoglycemia is the most common complication in GDM mothers, resulting from hyperinsulinemia in the fetus in response to maternal hyperglycemia in utero. After birth, the umbilical cord was clamped, the glucose supply was interrupted, and neonatal glucose concentrations decreased. Glucose is an essential metabolic component in newborns. Therefore, neonatal hypoglycemia might lead to poor long-term neurodevelopmental outcomes [37]. Furthermore, the mean neonatal birth weight, preterm birth, macrosomia, LGA, and rates of respiratory distress syndrome were not significantly different between pregnant women with abnormal 50 g GCTs without diagnoses of GDM and those with GDM. Therefore, we suggest that healthcare providers should carefully follow up on these groups of pregnant women.

The study’s limitations are as follows. The retrospective study design of this study might have resulted in selection bias, as participant selection was carried out by convenience sampling. The results were also subjected to confounding, as other risk factors that involve obstetric and neonatal outcomes might be present, but not measured. However, our study’s strengths were its large sample size and the fact that comprehensive data involving maternal and neonatal outcomes were gathered and analyzed. Additional studies should be performed prospectively to determine the maternal and neonatal outcomes of pregnant women with abnormal 50 g GCTs without diagnoses of GDM. Further studies to identify the risk factors for undesirable outcomes among these pregnant women are also suggested.

## 5. Conclusions

This study investigated the impact of an abnormal 50 g GCT during pregnancy without a diagnosis of GDM on maternal and neonatal outcomes in secondary care hospitals. The highest rate of birth asphyxia was found in pregnant women who had abnormal 50 g GCTs and one abnormal 100 g OGTT value, which was statistically higher than those in the abnormal 50 g GCT/normal 100 g OGTT and GDM groups. In addition, the mean neonatal birth weight and rates of preterm birth, macrosomia, LGA, and respiratory distress syndrome were not significantly different between pregnant women with abnormal 50 g GCTs without diagnoses of GDM and those with GDM. Therefore, this study suggests that healthcare providers should consider pregnant women with abnormal 50 g GCT tests without diagnoses of GDM as a high-risk group which should be closely monitored throughout pregnancy, until the postpartum period.

## Figures and Tables

**Table 1:** Characteristics of pregnant women with abnormal 50 g glucose challenge test from three hospitals (n = 1129).

Characteristics	Sichon Hospital n (%)	Thasala Hospital n (%)	Thungsong Hospital n (%)	Total n (%)
Number	373 (33.0%)	379 (33.6%)	377 (33.4%)	1129 (100.0%)
Age (years) ^a^	31 (26, 36)	31 (27, 35)	30 (26, 34)	31 (26, 35)
Age ≥ 25 years	308 (82.6%)	326 (86.0%)	312 (82.8%)	946 (83.8%)
Primigravida	116 (31.1%)	50 (13.2%)	88 (23.3%)	254 (22.5%)
Religion				
Buddhist	331 (88.7%)	228 (60.2%)	339 (89.9%)	898 (79.5%)
Islam	24 (6.5%)	139 (36.7%)	32 (8.5%)	196 (17.3%)
Christianity	18 (4.8%)	12 (3.2%)	6 (1.6%)	36 (3.2%)
History of GDM	4 (1.1%)	28 (7.4%)	16 (4.2%)	48 (4.2%)
History of macrosomia	13 (3.5%)	22 (5.8%)	8 (2.1%)	43 (3.8%)
History of DM in family	198 (53.1%)	148 (39.1%)	123 (32.6%)	469 (41.5%)
Total risks of GDM ^b^				
1 risk	164 (44.0%)	125 (33.0%)	184 (48.8%)	374 (41.9%)
>1 risk	209 (56.0%)	254 (67.0%)	193 (51.2%)	656 (58.1%)
Pre-pregnancy BMI (kg/m^2^) ^a^	25.5 (21.7, 29.9)	26.2 (22.5, 29.9)	22.4 (20.3, 25.2)	24.3 (21.2, 28.7)
Pre-pregnancy BMI ≥ 25 kg/m^2^	200 (53.6%)	213 (56.2%)	103 (27.3%)	516 (44.4%)
Maternal weight gain ^a^	13 (8, 17)	11 (8, 14)	11 (9, 15)	11 (8, 15)
Weight gain greater than recommendation ^c^	157 (42.1%)	121 (31.9%)	89 (23.6%)	367 (32.5%)
SBP at delivery (mmHg) ^a^	126 (117, 134)	126 (119, 137)	120 (110, 130)	124 (115, 134)
DBP at delivery (mmHg) ^a^	81 (75, 88)	80 (76, 89)	70 (70, 83)	79 (70, 87)
Mean 50 g GCT ^a^	162 (150, 180)	159 (149, 180)	160 (151.5, 176.8)	161 (150, 178)
GDM screening results				
Group 1: abnormal 50 g GCT, normal 100 g OGTT	121 (32.4%)	112 (29.6%)	164 (43.5%)	39 7(35.2%)
Group 2: abnormal 50 g GCT, one abnormal 100 g OGTT value	128 (34.3%)	149 (39.3%)	148 (39.3%)	425 (37.6%)
Group 3: GDM	124 (33.2%)	118 (31.1%)	65 (17.2%)	307 (27.2%)
Complete antenatal care visit ^d^	245 (65.7%)	215 (56.7%)	215 (57%)	675 (59.8%)

^a^ Data are presented as medians and inter-quartile ranges (IQRs). ^b^ Risk factors for GDM include age ≥ 30 years, pre-pregnancy body mass index (BMI) ≥ 25 kg/m^2^, family history of diabetes, presence of hypertension, previous GDM, and a history of fetal macrosomia, stillbirth, or fetal anomaly [4]. ^c^ Weight gain greater than recommendation is defined as total weight gain greater than 18 kg, 16 kg, 11.5 kg, and 9 kg for women with pre-pregnancy BMIs classified as underweight (<18.5 kg/m^2^), normal weight (18.5–24.9 kg/m^2^), overweight (25–29.9 kg/m^2^) and obese (≥30 kg/m^2^), respectively [24]. ^d^ Complete antenatal care visit is defined by the Ministry of Public health, Thailand as at least five visits, including at fewer than 12, 13–19, 20–25, 26–31, and 32–40 weeks of gestation.

**Table 2:** Maternal and neonatal outcomes of pregnant women with abnormal 50 g glucose challenge tests from three hospitals.

Outcomes	Sichon Hospital (n = 373)	Thasala Hospital (n = 379)	Thungsong Hospital (n = 377)	Total (n = 1129)
Maternal				
Cesarean delivery	231 (61.9%)	469 (44.6%)	173 (45.9%)	573 (50.8%)
Shoulder dystocia	6 (1.6%)	22 (5.8%)	10 (2.7%)	38 (3.4%)
Postpartum hemorrhage	13 (3.5%)	26 (6.9%)	8 (2.1%)	47 (4.2%)
Neonatal				
Prematurity	28 (7.5%)	22 (5.8%)	30 (8.0%)	80 (7.1%)
Birth weight (g) ^a^	3210 (2907.5, 3530)	3490 (3350, 3700)	3135 (2855, 3502.5)	3340 (2980, 3600)
Macrosomia	23 (6.2%)	28 (7.4%)	13 (3.4%)	64 (5.7%)
Large for gestational age	115 (30.8%)	194 (51.2%)	93 (24.7%)	402 (35.6%)
Respiratory distress syndrome	193 (51.7%)	88 (23.2%)	102 (27.1%)	383 (33.9%)
Hypoglycemia ^b^	104 (41.8%)	82 (31.5%)	46 (39.0%)	232 (37.0%)
Neonatal intensive care unit admission	106 (28.4%)	85 (22.4%)	78 (20.7%)	269 (23.8%)

^a^ Data are presented as medians and interquartile ranges (IQRs). ^b^ Total sample size of 627.

**Table 3:** Comparison of characteristics data among groups.

Characteristics	Group 1: Abnormal 50 g GCT, Normal 100 g OGTT (n = 397)	Group 2: Abnormal 50 g GCT, One Abnormal 100 g OGTT Value (n = 425)	Group 3: GDM (n = 307)	*p*-Value	
Age (years) ^a^	30 (26, 34)	31 (26, 35)	33 (29, 37)	<0.001	0.550 ^b^ <0.001 ^c^ <0.001 ^d^
Age >25 years	322 (81.1%)	339 (79.8%)	288 (92.8%)	<0.001	0.628 ^b^ <0.001 ^c^ <0.001 ^d^
Primigravida	93 (23.4%)	100 (23.5%)	61 (19.9%)	0.434	0.972 ^b^ 0.258 ^c^ 0.238 ^d^
Religion				0.648	0.526 ^b^ 0.535 ^c^ 0.590 ^d^
Buddhist	312 (78.6%)	345 (81.2%)	241 (78.5%)		
Islam	69 (17.4%)	68 (16%)	58 (18.9%)		
Christianity	16 (4%)	12 (2.8%)	8 (2.6%)		
Previous cesarean delivery	37 (9.3%)	47 (11.1%)	29 (9.4%)	0.658	0.411 ^b^ 0.955 ^c^ 0.480 ^d^
History of GDM	10 (2.5%)	15 (3.5%)	23 (7.5%)	0.003	0.399 ^b^ 0.002 ^c^ 0.017 ^d^
History of macrosomia	15 (3.8%)	16 (3.8%)	12 (3.9%)	0.994	0.992 ^b^ 0.929 ^c^ 0.920 ^d^
History of DM in family	17 (45.1%)	176 (41.4%)	114 (37.1%)	0.105	0.288 ^b^ 0.034 ^c^ 0.243 ^d^
Total number of risks of GDM				<0.001	0.788 ^b^ <0.001 ^c^ <0.001 ^d^
1 risk	184 (46.3%)	193 (45.4%)	96 (31.3%)		
>1 risk	213 (53.7%)	232 (54.6%)	211 (68.7%)		
Pre-pregnancy BMI (kg/m^2^) ^a^	23.79 (20.9, 28.1)	23.62 (20.8, 27.8)	26.34 (22.4, 30.2)	<0.001	0.946 ^b^ <0.001 ^c^ <0.001 ^d^
Pre-pregnancy BMI > 25 kg/m^2^	166 (41.8%)	171 (40.2%)	179 (58.3%)	<0.001	0.646 ^b^ <0.001 ^c^ <0.001 ^d^
Maternal weight gain (kg) ^a^	12 (9, 16)	12 (9, 16)	10 (7, 13)	<0.001	0.806 ^b^ <0.001 ^c^ <0.001 ^d^
Total weight gain greater than recommendation	138 (34.8%)	144 (33.9%)	85 (27.7%)	0.144	0.320 ^b^ 0.076 ^c^ 0.192 ^d^
Mean 50 g GCT ^a^	159 (149, 179)	160 (149–179)	164 (152–185)	0.096	0.406 ^b^ 0.032 ^c^ 0.156 ^d^
SBP at delivery (mmHg) ^a^	123 (110, 134)	123 (113, 133.5)	125 (118, 135)	0.013	0.402 ^b^ 0.004 ^c^ 0.032 ^d^
DBP at delivery (mmHg) ^a^	79 (70, 87.5)	79 (70, 87)	80 (72, 88)	0.090	0.560 ^b^ 0.032 ^c^ 0.105 ^d^
Complete antenatal care visit	237 (59.7%)	233 (54.8%)	205 (66.8%)	0.005	0.158 ^b^ 0.054 ^c^ 0.001 ^d^

^a^ Data are presented as medians and interquartile ranges (IQRs). ^b^ Comparison of groups 1 and 2. ^c^ Comparison of groups 1 and 3. ^d^ Comparison of groups 2 and 3.

**Table 4:** Comparison of maternal and neonatal outcomes among groups.

Outcomes	Group 1: Abnormal 50 g GCT, Normal 100 g OGTT (n = 397)	Group 2: Abnormal 50 g GCT, One Abnormal 100 g OGTT Value (n = 425)	Group 3: GDM (n = 307)	*p*-Value	
Maternal					
Cesarean delivery	173 (43.6%)	210 (49.4%)	190 (61.9%)	<0.001	0.125 ^b^ 0.001 ^c^ 0.003 ^d^
Shoulder dystocia	18 (4.5%)	13 (3.1%)	7 (2.3%)	0.234	0.267 ^b^ 0.109 ^c^ 0.524 ^d^
Postpartum hemorrhage	20 (5%)	20 (4.7%)	7 (2.3%)	0.149	0.825 ^b^ 0.059 ^c^ 0.086 ^d^
Neonatal					
Prematurity	24 (6%)	28 (6.6%)	28 (9.1%)	0.254	0.479 ^b^ 0.122 ^c^ 0.203 ^d^
Birth weight (g) ^a^	3350 (2990, 3600)	3325 (2957, 3600)	3345 (3005, 3590)	0.763	0.545 ^b^ 0.944 ^c^ 0.517 ^d^
Macrosomia	18 (4.5%)	26 (6.1%)	20 (6.5%)	0.466	0.313 ^b^ 0.249 ^c^ 0.827 ^d^
Large for gestational age	134 (33.8%)	144 (33.9%)	124 (40.4%)	0.122	0.969 ^b^ 0.070 ^c^ 0.071 ^d^
Respiratory distress syndrome	129 (32.5%)	140 (32.9%)	114 (37.1%)	0.376	0.891 ^b^ 0.199 ^c^ 0.240 ^d^
Birth asphyxia	7 (1.8%)	25(5.9%)	10 (3.3%)	0.007	0.002 ^b^ 0.200 ^c^ 0.100 ^d^
Hypoglycemia ^e^	58 (36.9%)	54 (28.4%)	120 (42.9%)	<0.001	0.091 ^b^ 0.227 ^c^ 0.001 ^d^
Neonatal intensive care unit admission	73 (18.4%)	87 (20.5%)	109 (35.5%)	<0.001	0.451 ^b^ <0.001 ^c^ <0.001 ^d^

^a^ Data are presented as medians and inter-quartile ranges (IQRs). ^b^ Comparison of group 1 and group 2. ^c^ Comparison of group 1 and group 3. ^d^ Comparison of group 2 and group 3. ^e^ Total sample size of 627.

## Data Availability

Data will be made available upon request.
